# Bronchiolitis in children ≤ 12 months during the pre-flu season of 2024: A case series

**DOI:** 10.4102/safp.v67i1.6109

**Published:** 2025-04-17

**Authors:** Marietjie Brits, Hanneke Brits

**Affiliations:** 1 Department of Family Medicine, Faculty of Health Sciences, University of the Free State, Bloemfontein, South Africa

**Keywords:** bronchiolitis, respiratory distress, RSV, Influenza A, treatment

## Abstract

**Background:**

In March 2024, the National Institute for Communicable Diseases (NICD) warned against a respiratory syncytial virus (RSV) outbreak during the pre-flu season, 2024. Globally, RSV is responsible for most respiratory infections in young children. Since April 2024, we have noticed a more severe presentation of children with bronchiolitis at the National District Hospital, Bloemfontein. We aimed to investigate the causative organism/s and the appropriateness of the treatment guidelines in our setting.

**Methods:**

A retrospective file review was carried out as part of the normal audit process. All children admitted with bronchiolitis during the 4-week study period were included in the case series. Ethical approval was obtained from relevant authorities.

**Results:**

Ten children ≤ 12 months presented with bronchiolitis symptoms. The Modified Tal Score was measured, with values between 8 and 12, indicating moderate and severe bronchiolitis in all cases. The demographics regarding gender, nutrition and vaccination status did not differ from previous years. Influenza A was identified in 60% of the cases, with RSV in only 20% of the cases. All the children responded well to symptomatic treatment and recovered.

**Conclusion:**

Most bronchiolitis cases were caused not by the predicted RSV outbreak but by Influenza A.

**Contribution:**

Despite presenting with severe symptoms, all the children responded to symptomatic treatment as set out in our guidelines.

## Introduction

In March 2024, the National Institute for Communicable Disease (NICD) warned against a respiratory syncytial virus (RSV) outbreak during the pre-flu season of 2024.^1^ In South Africa, RSV usually peaks in week 17, and the flu season starts in week 19 of the calendar year.^2^ Therefore, we anticipated the RSV outbreak to be between the middle of April and the middle of May 2024. Respiratory syncytial virus is responsible for most respiratory infections in young children globally^3^ and locally,^1^ while many small children present with bronchiolitis. Respiratory syncytial virus is the second most common cause of respiratory death in children under 1 year of age.^4^ This warning warranted us to review the bronchiolitis guidelines in our setting.

The literature shows that RSV is the most common factor in bronchiolitis, contributing to between 30% and 80% of cases.^5,6^ Other viruses, such as human metapneumovirus, rhinovirus, adenovirus and parainfluenza virus, contribute between 7% and 8%, and Influenza virus 2%.^5^

Bronchiolitis is a clinical diagnosis, and the definition may vary slightly in different textbooks and research articles, for example, some use a cut-off of 12 months, while others use 24 months.^7^ Most definitions include a viral upper respiratory tract infection with rhinorrhoea. This is followed by a lower respiratory tract infection, with or without wheezes, persistent coughing and respiratory distress. Fever may be present.^7,8^

The National District Hospital is part of the Academic Training Complex of the University of the Free State. The ward had 21 primary health care paediatric beds and was the only 24-h walk-in paediatric service in the catchment area of Bloemfontein. The service manages around 250 consultations and 40 admissions a month. During previous years, between five and six children were admitted per month during the peak bronchiolitis season.

The mainstay of bronchiolitis treatment is supportive, which include hydration, feeding and oxygen.^9,10^ In our setting, a room air saturation of < 92% warrants admission and nasal oxygen. The rest of the management is symptom dependent. Fever is treated with paracetamol, primarily to relieve discomfort.^9^ In children with a high fever and severe distress, a STAT dosage of broad-spectrum antibiotics is administered intravenously. If the c-reactive protein (CRP) result returns ≤ 40 and no specific bacterial infection is diagnosed, no further antibiotic should be prescribed.^10^ Hypertonic saline nebulisation is prescribed for wheezing.^11^ Although oral steroids are not part of the routine treatment of bronchiolitis, we prescribe short courses to admitted children with persistent wheezing after 4 h or wheezing not improving after two nebulisations. This practice is, however, from clinical experience and limited scientific evidence.^12,13^ Antiviral treatment is not part of the national paediatric treatment guidelines.^9^ The facility has protocols for laboratory confirmation of organisms when atypical patient presentations, outbreaks or abnormal trends are detected.

In April 2024, the number of small children presenting to the National District Hospital (NDH) with bronchiolitis increased. This was expected after the NICD warning and the start of the pre-flu season.^1^ We thought it was the usual bronchiolitis caused by RSV, but the clinical pictures did not fit. Clinically, the children were more distressed with low oxygen saturation levels.

### Question

Why did we see an increase and atypical presentation of bronchiolitis in under-1-year-old children at NDH, and did we manage them appropriately?

## Method

A retrospective, descriptive case series was conducted.

### Study population and sample

The study population consisted of all admitted children ≤ 12 months of age who presented to NDH during the pre-flu season, weeks 16–19 (15 April 2024 – 12 May 2024), with bronchiolitis symptoms. No sampling took place, and all 10 children were included. All 10 children received respiratory viral panel swabs as part of the atypical presentation cohort.

### Data collection

The researchers collected retrospective data from patient files on Research Electronic Data Capture (REDCap). The data included age, vaccination status, nutritional status, presenting symptoms, vital signs on admission, oxygen saturation, treatment received, blood results, viral panel results and outcome. No pilot study was performed as the results were part of the normal audit process performed in the hospital.

### Data analysis

The researchers analysed the data directly on REDCap. Research Electronic Data Capture is a University of the Free State-approved secure web application for managing online databases and surveys.^14^ The severity of bronchiolitis for each patient was calculated with the Modified Tal Score (MTS). The maximum score is 12, with ≤ 5 classified as mild, 6–10 as moderate and 11–12 as severe.^15^

## Management and outcome

All 10 children ≤ 12 months of age who presented with respiratory distress during the pre-flu season of 2024 were included in the case series.

The ages varied between 3 months and 12 months, with a median of 6 months. There were no differences in gender distribution. None of the children was born prematurely or had chronic underlying diseases. Most children (*n* = 7/10) plotted as normal weight-for-age, while two were severely overweight and one was underweight. In one child, the vaccination status was not up to date. In two cases, there was evidence of passive smoke exposure from parents or caretakers. In Table 1, results for the different cases are displayed.

**TABLE 1:** Results of the case series.

Case	Admission week	Age in months	Respiratory rate per min	Temperature in °C	% O_2_ saturation in room air	Modified Tal score	Nutritional status	White cell count	C-reactive protein	Respiratory viral swab – PCR	Co-infection	Length of stay in days (LoS)
1	16	11	50	39.3	88	9	Severely underweight	2.84	39	Human metapneumovirus	-	12
2	16	12	46	38.6	88	8	Normal weight	8.03	2	Influenza A	-	4
3	17	3	62	36.1	87	9	Severely overweight	4.55	1	Influenza A	-	2
4	17	12	48	38.6	85	8	Normal weight	17.66	51	Influenza A	-	3
5	17	9	58	38.0	67	11	Normal weight	7.54	2	Influenza A	-	5
6	17	6	74	36.1	86	11	Normal weight	12.46	4	RSV	Human Rhino	5
7	17	7	64	36.3	71	10	Normal weight	21.08	128	Influenza A	Human Rhino	5
8	18	4	58	38.8	90	12	Severely overweight	7.37	1	COVID-19	-	5
9	18	8	20	38.2	90	10	Normal weight	12.80	11	RSV	-	2
10	19	10	66	38.1	81	10	Normal weight	14.65	18	Influenza A	-	5

COVID-19, coronavirus disease 2019; RSV, respiratory syncytial virus; PCR, polymerase chain reaction.

Clinically, all the children presented with saturation levels < 92% on room air, tachypnoea, wheezing and hyperinflation on chest X-rays, while 70% had a temperature above 38 °C.

All children were classified as moderate to severe bronchiolitis using the calculated MTS. The scores varied between 8 and 12, with a median and mean of 10. The child with the MTS of 12 was severely overweight and tested positive for coronavirus disease 2019 (COVID-19) (Case 8).

The laboratory results indicated bacterial infection in three children, two with leucocytosis and one with leukopenia. All three children had thrombocytosis, while the two with leucocytosis had CRP levels of 51 and 128 (Case 4 and Case 7), respectively, and the one with leukopenia had a CRP level of 39 (Case 1).

The respiratory panel nasal swabs (PCR testing) for all children were positive. Sixty per cent tested positive for Influenza A and 20% tested positive for RSV. Two patients, one with Influenza A (Case 7) and one with RSV (Case 6), had a co-infection with human rhinovirus.

Regarding management, all children received nasal-prong oxygen for saturation levels ≤ 92% and hypertonic saline nebulisation for wheezing. Seventy per cent received paracetamol for temperatures ≥ 38.0 °C. The three children with possible bacterial infections received a full course of antibiotics, and 60% of children received a 5-day course of oral steroids for continuous wheezing. None of the children deteriorated during admission. All the children recovered well, with an average length of stay (LoS) of 5 days. The LoS varied between 2 days and 12 days (Case 1).

## Discussion

The demographic profile of admitted children correlated with previous observations and unpublished data in the current setting with an equal gender distribution, mostly well-nourished children and up-to-date vaccination status. The 10 bronchiolitis admissions during the study period were higher than that in the previous years (during the same study period as recorded in hospital statistics), which varied between five and six. In studies with comparable populations, there was a slight male dominance.^16,17^ Our population did not have known risk factors for severe bronchiolitis such as prematurity, underlying diseases and poor vaccination status. Passive smoking was present in two cases.^16,18^ However, our numbers are too small to make any deductions. The small numbers and short study period are acknowledged as a study limitation, and further research in our setting is suggested.

The MTS scores of all the admitted children were in the high moderate (≥ 8) or severe category. In our setting, we have a low threshold for admission and would admit even children with MTS scores as low as five. However, none of the children who presented with bronchiolitis had low scores, which may indicate that the children presented with more severe symptoms. Compared with a study that correlates different severity scores, the mean presenting scores using three different scoring systems were all in the mild category.^8^ This made us believe that the presentation differed from previous years.

All the children received symptomatic treatment according to our current guidelines. The three children with evidence of bacterial infections received a full course of antibiotics, and those with continuous wheezing received a 5-day course of oral prednisone. None of the children deteriorated during admission, and all were discharged within 2 days – 12 days, with an average LoS of 5 days. De Rose et al. reported an average LoS for more severe bronchiolitis as 6 days.^8^ Therefore, the current treatment guidelines were appropriate for managing children with moderate to severe bronchiolitis in our primary healthcare setting.

The results of viral swabs to identify organisms did not align with previous studies. We identified viruses in 100% of the swabs. In other studies, viral swabs could only identify viruses in 45% – 57% of cases.^16,18^ This may be because the treating doctors were well trained and participated in previous studies using nasal swabs to identify organisms causing COVID-19 and pertussis. Contrary to previous studies^4,5,16,18^ and the NICD warning,^1^ Influenza A was the most prevalent organism (60%), with RSV contributing to only 20% of cases. During the study period, the results from the NICD reflected a dominance of RSV in weeks 16 cases and 17, with 48 cases and 58 cases, respectively, compared to 16 cases and 32 cases of Influenza A. During week 19, the Influenza A cases increased to 76 compared with 49 RSV cases.^2^ This result was not expected as influenza is a vaccine-preventable disease.^19^

In a severely ill, malnourished child (Case 1), human metapneumovirus was identified. The Centres for Disease Control (CDC) overview on human metapneumovirus states that it usually causes mild infections, except in very young children, immunocompromised or elderly people. The treatment for human metapneumovirus is symptomatic.^20^ The child who tested positive for COVID-19 was severely overweight, with an MTS score of 12. The child recovered within 3 days of symptomatic treatment.

### What we knew

The NICD predicted an RSV outbreak during the pre-flu season of 2024. Respiratory syncytial virus is the #1 pathogen responsible for bronchiolitis.

### What we learned

- Influenza A was the most prevalent organism causing bronchiolitis during the pre-flu season 2024 in the National District Hospital.
- The children presented with more severe symptoms.
- Irrespective of the organism, all the children responded to the symptomatic treatment as set out in the treatment guidelines.

### What we want to recommend

- Influenza vaccines for children older than 6 months, their immediate family and pregnant mothers may be considered if these results can be confirmed in further studies. This recommendation is in line with the World Health Organization (WHO) guidelines on flu vaccines.^19^ In the 2024, NICD guidelines on influenza, pregnant women are prioritised to receive vaccines.^21^
- Implementing preventative measures to curb the spread of viruses, including hand washing, hygienic cough techniques and possible vaccination, may prevent future bronchiolitis outbreaks.

## References

[1] National Institute for Communicable Disease. NICD. Start of the Respiratory Syncytial Virus (RSV) season: Alert to clinicians [homepage on the Internet]. 2024 [cited 2024 Nov]. Available from: https://www.nicd.ac.za/start-of-the-respiratory-syncytial-virus-rsv-season-alert-to-clinicians/#:~:text=The%20RSV%20season%20usually%20precedes,(excluding%202020%20and%202021).

[2] National Institute for Communicable Disease. NICD weekly respiratory pathogens surveillance report [homepage on the Internet]. 2024 [cited 2024 Nov]. Available from: https://www.nicd.ac.za/diseases-a-z-index/disease-index-covid-19/surveillance-reports/weekly-respiratory-pathogens-surveillance-report-week/

[3] Barbati F, Moriondo M, Pisano L, et al. Epidemiology of respiratory syncytial virus-related hospitalization over a 5-year period in Italy: Evaluation of seasonality and age distribution before vaccine introduction. Vaccines (Basel). 2020;8(1):15. 10.3390/vaccines8010015 31947976 PMC7157234

[4] Azzari C, Baraldi E, Bonanni P. Epidemiology and prevention of respiratory syncytial virus infections in children in Italy. Ital J Pediatr. 2021;47(1):198. 10.1186/s13052-021-01148-8 34600591 PMC8487331

[5] Von Mollendorf C, Berger D, Gwee A, et al. Aetiology of childhood pneumonia in low- and middle-income countries in the era of vaccination: A systematic review. J Glob Health. 2022;12:10009. 10.7189/jogh.12.10009 35866332 PMC9305023

[6] Erickson EN, Bhakta RT, Mendez MD. Pediatric bronchiolitis [homepage on the Internet]. StatPearls; 2023 [cited 2024 June]. Available from: https://www.ncbi.nlm.nih.gov/books/NBK519506/#:~:text=Introduction-,Bronchiolitis%20is%20inflammation%20of%20the%20bronchioles%20usually%20caused%20by%20an,and%20admissions%20to%20the%20hospital 30137791

[7] Dalziel SR, Haskell L, O’Brien S, et al. Bronchiolitis. Lancet. 2022;400(10349):392–406. 10.1016/S0140-6736(22)01016-9 35785792

[8] De Rose DU, Maddaloni C, Martini L, Braguglia A, Dotta A, Auriti C. Comparison of three clinical scoring tools for bronchiolitis to predict the need for respiratory support and length of stay in neonates and infants up to three months of age. Front Pediatr. 2023;11:1040354. 10.3389/fped.2023.1040354 36873647 PMC9983816

[9] Standard treatment guidelines and essential drug list [homepage on the Internet]. National Department of Health. Hospital-Level, Paediatric; 2024 [cited 2024 June]. Available from: https://knowledgehub.health.gov.za

[10] Dangor Z, Lala SG, Verwey C, et al. Bronchiolitis v. bronchopneumonia: Navigating antibiotic use within the lower respiratory tract infection spectrum. S Afr Med J. 2023;113(6):e709. 10.7196/SAMJ.2023.v112iX.709 37278266

[11] Zhang L, Mendoza-Sassi RA, Wainwright CE, Aregbesola A, Klassen TP. What are the benefits and risks of hypertonic saline solution via nebuliser for treating infants with acute bronchiolitis, compared to normal saline solution? [homepage on the Internet]. Cochrane. 2023. Available from: https://www.cochrane.org/CD006458/ARI_what-are-benefits-and-risks-hypertonic-saline-solution-nebuliser-treating-infants-acute

[12] Csonka PE, Kaila M, Laippala P, Iso-Mustaja M, Vesikari T, Ashorn P. Oral prednisolone in the acute management of children aged 6 to 30 months with viral respiratory infection induced lower airway disease: Randomised, placebo-controlled trial. J Pediatr. 2003;143(6):725–730. 10.1067/S0022-3476(03)00498-0 14657816

[13] Garrison MM, Christakis DA, Harvey E, Cummings P, Davis RL. Systemic corticosteroids in infant bronchiolitis: A meta-analysis. Pediatrics. 2000;105(4):e44. 10.1542/peds.105.4.e44 10742365

[14] REDCap 2024 [homepage on the Internet]. [cited 2024 June]. Available from https://projectredcap.org/

[15] Golan-Tripto I, Goldbart A, Akel K, Dizitzer Y, Novack V, Tal A. Modified tal score: Validated score for prediction of bronchiolitis severity. Pediatr Pulmonol. 2018;53(6):796–801. 10.1002/ppul.24007 29655288

[16] Kulhalli P, Dakshayini JN, Ratageri VH, Shivanand I, Wari PK. Risk factors for bronchiolitis. J Pediatr Crit Care. 2020;7(2):79–83. 10.4103/JPCC.JPCC_23_20

[17] Petrarca L, Nenna R, Di Mattia G, et al. Bronchiolitis phenotypes identified by latent class analysis may influence the occurrence of respiratory sequelae. Pediatr Pulmonol. 2022;57(3):616–622. 10.1002/ppul.25799 34931488

[18] Manti S, Staiano A, Orfeo L, et al. UPDATE – 2022 Italian guidelines on the management of bronchiolitis in infants. Ital J Pediatr. 2023;49:19. 10.1186/s13052-022-01392-6 36765418 PMC9912214

[19] WHO. World Health Organisation. Influenza (Seasonal) [homepage on the Internet]. 2023 [cited 2024 Nov]. Available from: https://www.who.int/news-room/fact-sheets/detail/influenza-(seasonal)

[20] CDC. Human Metapneumovirus [homepage on the Internet]. Centres for Disease Control and Prevention; 2023 [cited 2024 Nov]. Available from: https://www.cdc.gov/human-metapneumovirus/about/index.html

[21] National Institute for Communicable Disease. Influenza: NICD recommendations for the diagnosis, management, prevention and public health response [homepage on the Internet]. 2024 [cited 2024 Nov]. Available form: https://www.nicd.ac.za/wp-content/uploads/2024/05/Influenza-guidelines-2024_final.pdf

